# INVESTIGATING LINKS BETWEEN CHILDHOOD HEALTH AND FAMILY LIFE AND ACCELERATED BIOLOGICAL AGING

**DOI:** 10.1093/geroni/igac059.1750

**Published:** 2022-12-20

**Authors:** Marina Larkina, Jacqui Smith

**Affiliations:** University of Michigan, Ann Arbor, Michigan, United States; University of Michigan, Ann Arbor, Michigan, United States

## Abstract

An epigenetic clock measure, DNA methylation (DNAm) PhenoAge, differentiates individual aging trajectories and has been shown to predict mortality and morbidities. Despite suggestions that early-life experiences may shape epigenetic aging, little is known to date about specific risk and protective factors. We use data from Health and Retirement Study (HRS) to investigate the role of childhood factors, including health and family financial situation, in epigenetic age acceleration. The sample (N = 3952, M age = 64, range 50-100) included participants from the HRS 2016 Venous Blood Study and those, who reported about their childhood health and family. Using logistic regression, we predicted DNAm PhenoAge acceleration, calculated as the residuals resulting from regressing DNAm PhenoAge on chronological age and coded as 0 or 1 (1 = positive values, faster epigenetic aging rate). Participants with more years of education were less likely to have accelerated epigenetic aging (OR: 0.963, 95%CI[0.941-0.985], p < .001). However, self-reported chronic illnesses before age 16 (15 possible conditions), self-rated childhood health, and family financial situation before age 16 were not associated with accelerated aging. In addition, men had higher likelihood of accelerated aging than women (OR: 1.145, 95%CI[1.007-1.301], p < .05). Race/ethnicity and age cohort (e.g., being younger or older age 65) were not significant predictors. Our results highlight that investigation of relation between childhood disadvantages and DNAm PhenoAge acceleration might need to include other indicators (e.g., residential history). Future work is needed also to identify life course moderators of the clock efficacy.

